# Reduced genetic diversity and alteration of gene flow in a fiddler crab due to mangrove degradation

**DOI:** 10.1371/journal.pone.0182987

**Published:** 2017-08-24

**Authors:** Alex Nehemia, Marc Kochzius

**Affiliations:** 1 Marine Biology, Ecology and Biodiversity, Vrije Universiteit Brussel (VUB), Pleinlaan 2, Brussels, Belgium; 2 Department of Biological Sciences, Sokoine University of Agriculture, Morogoro, Tanzania; National Cheng Kung University, TAIWAN

## Abstract

The fiddler crab *Austruca occidentalis is* a dominant species in mangrove forests along the East African coast. It enhances soil aeration and, through its engineering activities, makes otherwise-inaccessible food available for other marine organisms. Despite its importance, the habitat of *A*. *occidentalis* is threatened by human activities. Clearing the mangroves for salt farming and selective logging of mangroves trees continue to jeopardise mangrove ecosystems in the Western Indian Ocean. This study aims to use partial mitochondrial COI gene sequences and nuclear microsatellites to determine whether salt farming activities in mangroves have a negative impact on the genetic diversity and gene flow of *A*. *occidentalis* collected along the Tanzania coast. The level of genetic diversity for both mitochondrial DNA and nuclear microsatellites are relatively lower in samples from salt ponds compared to natural mangrove sites. Analysis of molecular variance (AMOVA) among all populations showed low but significant differentiation (COI: F_st_ = 0.022, P < 0.05; microsatellites: F_st_ = 0.022, P < 0.001). A hierarchical AMOVA indicates lower but significant genetic differentiation among populations from salt ponds and natural mangroves sites (COI: F_ct_ = 0.033, P < 0.05; microsatellites: F_ct_ = 0.018, P = < 0.01). These results indicate that salt farming has a significant negative impact on the genetic diversity of *A*. *occidentalis*. Since higher genetic diversity contributes to a stable population, restoring the cleared habitats might be the most effective measures for the conservation of genetic diversity and hence adaptive potential to environmental change in this species.

## Introduction

### Mangroves and salt farming

The loss of mangroves continues to increase rapidly in developing countries, where more than 90% of the world’s mangroves are located [1]. In developing countries along the East African coast of the Western Indian Ocean (WIO) extensive areas of mangroves have been cleared to pave a way for the construction of salt ponds [2–4]. Salt farming in mangroves is among the activities that contribute to the loss of mangroves globally [5–6]. This economic activity continues to be a threat for mangroves, which are estimated to have been reduced by 35 to 50% within the last 50 years mainly due to anthropogenic activities [7].

Salt production in mangroves ignores the important function of mangroves as habitat and nursery ground for many marine and terrestrial fauna [8, 9]. Salt production involves clear-cutting of extensive areas of mangroves and selective logging of mangrove trees for firewood, as well as construction of dykes around the salt ponds and water reservoirs. In addition, mangrove trees are selectively logged for building huts used to store salt and as houses for people involved in these activities [10]. Loss of mangroves in the area results in the loss of habitats for important marine species, such as crabs and snails that depend on mangroves for their survival. Removal of a considerable part of natural vegetation affects the mangrove ecosystem by reducing the habitat complexity and altering the drainage pattern and run-off in the area [11].

Tanzania is one of the East African countries at the WIO with a mangrove area that covers about 1,587.44 Km^2^ [12] and all of them are classified as forest reserves [3]. This ecosystem is threatened by salt farming [11, 13–14], which is legal and takes place in most mangroves at the Tanzanian coast. Some salt pans have been abandoned but still retain the dykes that prevent free tidal flow in the area.

### Impacts and indicators of ecological disturbances

Ecological disturbance cause habitat disturbances once it changes food availability and predation [15–16]. Consequently this reduces the population size and genetic diversity [17]. For example, habitat disturbances through establishment of artificial structures in natural marine habitats were observed to cause lower genetic diversity of the limpet *Patella caerulea* [18]. The reduced genetic diversity decreases the capability of a species to adapt to environmental disturbances and can cause decreases in its long-term survival [19–21].

The fiddler crab *Austruca occidentalis* has been found to be a good indicator of negative impacts of anthropogenic activities, such as sewage discharge in the mangrove ecosystem [22]. This species is well known for its engineering activities through burrowing that promote nutrient cycling in mangrove ecosystems [23–25]. They act as a link between microbial production and higher trophic levels through conversion of a large part of the bacterial production into food items for larger predators [26]. This species is the most dominant of all *Austruca sp*. occurring at the intertidal shores of Eastern Africa [27]. The male is characterised by the asymmetry of the chelipeds, with the larger claw used in courtship display to attract females and fight with other males [28]. It has also a minor claw used for gathering food, which is similar to those of females [29]. In the salt ponds area it is often observed in groups during daytime, sometimes sharing the habitat with *Cranuca inversa* (field observation).

The pelagic larval duration of *A*. *occidentalis* larvae is about 28 days and permits extensive gene flow along the East African coast [30]. Planktonic dispersal plays an important role in homogenising gene frequencies, and the lack of larval exchange is thought to increase genetic differentiation [31]. Despite potentially high gene flow in marine species, human disturbance, larval characteristics, such as behaviour and physiological requirements, can interrupt natural dispersal, which is necessary to maintain genetic variability [30, 32]. It has been shown that even species that have relatively high gene flow, such as *A*. *occidentalis*, may suffer from habitat fragmentation, resulting to genetic fragmentation between isolated populations [33]. Understanding the genetic diversity among and within populations is necessary for efficient management of natural living resources.

### Molecular markers and habitat disturbances

The use of molecular markers in population genetics studies has proven to be important in shedding light on several aspects related to the biology, ecology and evolution of many organisms [34]. Molecular markers can be used to provide information for conservation of species, because they can be used to assess the impact of habitat destruction on genetic diversity. Therefore, molecular markers can provide baseline data for long-term conservation plans [35]. Due to the threat of on-going degradation, genetic information is required for effective management of marine species in the WIO [36].

Both mitochondrial DNA (mtDNA) and nuclear microsatellite data are helpful in analysing the genetic population structure of marine species [37–39]. However, given the low resolution of mitochondrial cytochrome oxidase subunit 1 (COI) sequences in detecting recent loss of genetic variation and its maternal inheritance [40], sensitive molecular markers with higher polymorphism and more power in detecting recent traces of gene flow fluctuations, such as microsatellites, are more suitable. In this study, we used both COI sequences and microsatellites to assess if salt production in mangroves has a negative impact on the genetic diversity and demography of *A*. *occidentalis*. Conservation of species can be improved by investigating factors that can lead to evolutionary change over a short time period [41].

The objectives of this study were to determine: 1) if salt farming affects the genetic diversity of *A*. *occidentalis* through inbreeding and bottleneck effects; 2) if salt farming has negative effects on the effective population size; and 3) if dispersal patterns of this species have been altered by salt farming activities. To achieve this, we assessed the degree of genetic variation and structure in populations from mangroves at salt ponds and natural mangroves. Then we examined the levels of heterozygosity and estimated effective population sizes, inbreeding and migration rates. We finally compared the genetic diversity and demographic indices between the populations at salt ponds and natural mangroves.

## Material and method

### Sampling

No specific permissions were required for these locations/activities. The Tanzanians students are allowed by the government to conduct the scientific research but in order to export the samples abroad for laboratory analysis, the special licence/permit that is provided by the Ministry of Agriculture, Livestock and Fisheries (Fisheries development division-HQ, Dar es Salaam) of the united republic of Tanzania is required. The code number of the special permit for exporting our samples to Belgium at Free university of Belgium (VUB) was 1997/2015. The field studies did not involve endangered or protected species.

**Fig 1:**
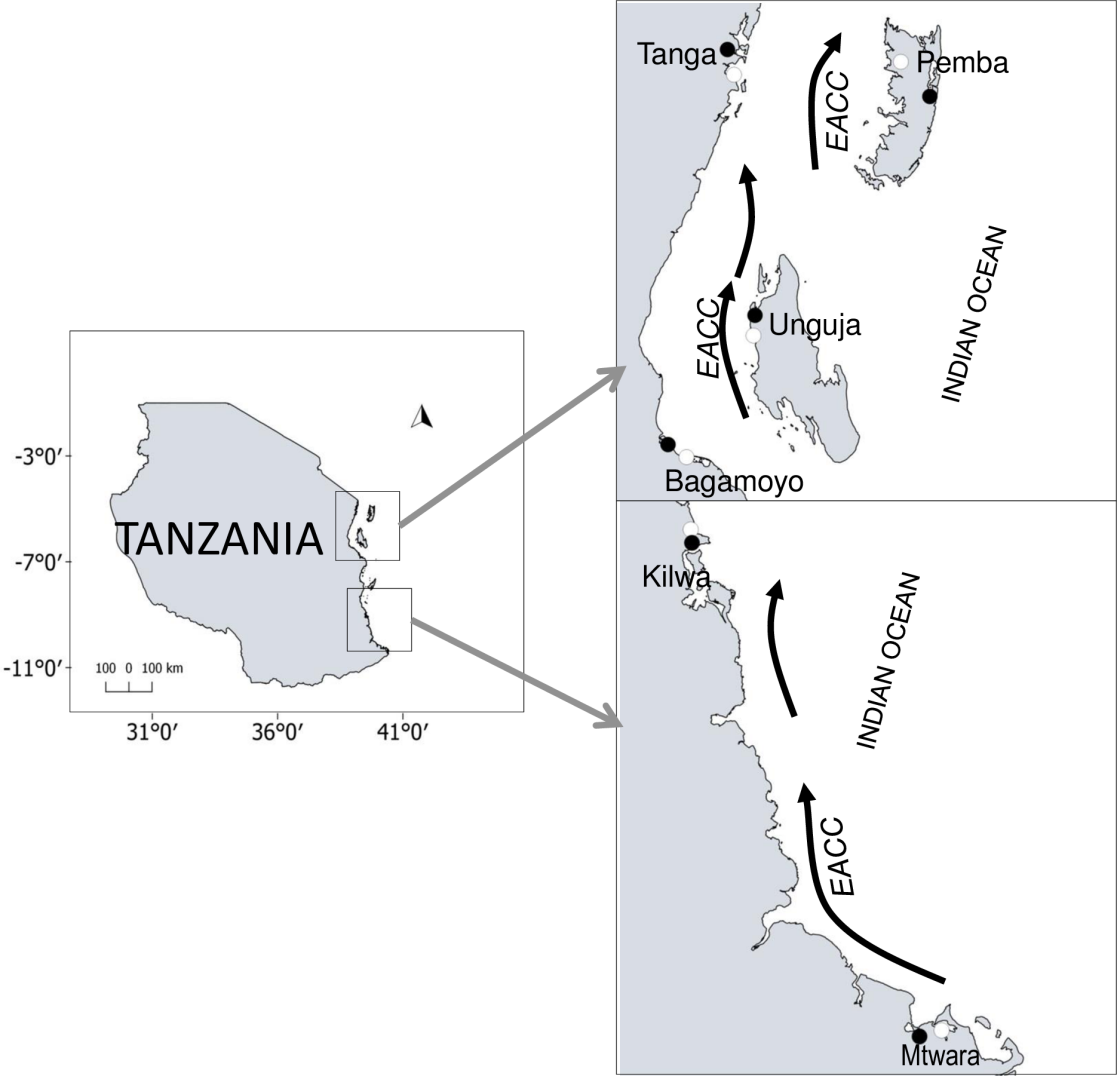
Map showing sample sites of the fiddler crab *Austruca occidentalis* from the Western Indian Ocean (partial mitochondria cytochrome oxidase subunit I (COI) sequences and microsatellites). The white filled circles represent natural mangroves and black filled circles represent mangroves at salt ponds. The East African Coast Current (EACC) is indicated by black arrows.

Samples of *A*. *occidentalis* were collected at 12 sites, of which six sites are from mangroves at salt ponds and the rest are from natural mangroves (Fig 1). The locations of the sites (in the bracket Latitudes, Longitude) for mangroves at salt ponds are Tanga–Mpirani (4° 58.54' S, 39° 06.04' E), Bagamoyo–Nunge (6° 24.80' S, 38° 53.08' E), Kilwa–Makubuli (8° 55.53' S, 39° 30.44' E), Mtwara–Kilimahewa (10° 17.60 'S, 40°09.10' E), Pemba–Wete (5° 08.76' S, 39°50.14' E) and Unguja–Makoba (5° 56.57' S, 39°12.06' E). For natural mangroves are Tanga–Lumbachia (5° 04.05' S, 39° 07.46' E), Bagamoyo–Kaole (6° 27.54' S, 38° 57.11' E), Kilwa–Timaki (8° 53.53' S, 39° 30.44' E), Mtwara–Ngw’ale (10° 16.33' S, 40° 12.85' E), Pemba–Chakechake (5° 01.21' S, 39° 43.82' E) and Unguja–Fujoni (6° 1' S, 39°11.73' E). Sampling was conducted during low tide on the landside of mangroves at salt ponds and in natural mangroves between July and August 2015. For each site of mangroves at a salt pond sampled, a natural mangrove site not far from it was also sampled. The longest distance recorded between the salt pond and a natural mangrove site was (82 km) in Pemba, but for all other sites it ranged from 4 to 18 km. The dominant prevailing ocean current in this region is the East African Coast Current (EACC) (Fig 1), which flows northward throughout the year. It originates from the South Equatorial Current (SEC), which flows from East to West throughout the year at around 12°S latitude [42]. Narrow streams of strong currents have also been reported to flow northwards parallel to the coast in the Mafia, Zanzibar, and Pemba Channels [43].

### DNA extraction

Tissue samples from a segment of the last pereiopod of *A*. *occidentalis* were collected and immediately preserved in 99% ethanol. Total DNA was isolated for mitochondrial DNA (mtDNA) and microsatellite analyses using either the QIAGEN (Düsseldorf, Germany) or E.Z.N.A.^®^ Tissue DNA Kit (Omega Bio-Tek, California, USA) extraction kit. The extracted DNA was visualised in 2% TBE agarose gels.

### Amplification of mtDNA and sequencing

The primer jgLCO1490 (5’-TITCIACIAAYCAYAARGAYATTGG-3’) and jgHCO2198 (5’-TAIACYTCIGGRTGICCRAARAAYCA-3’) [44] was used to amplify a portion of the COI gene. PCR was performed in a BIORAD T100^TM^ thermocycler in a 50 μl reaction volume containing 0.2 mM dNTPs, 1 mM 10x PCR reaction buffer, 0.2 μM primers primer, 2.5 μl of 10 mg/ml BSA, 3 mM MgCl_2_, 3 μl DNA extract, and 1 U Taq polymerase. The temperature profile was set at 94°C for 5 minutes as initial denaturation, followed by 35 cycles of denaturation at 94°C for 60 seconds, annealing at 50°C for 1.5 minutes, 72°C of extension for 1 minute and 72°C of final extension for 5 minutes. PCR products were electrophoresed and visualised by using GelRed in 2% TBE agarose gels. An ABI 3770XL automated sequencer (Applied Biosystems, Foster City, USA) was used for sequencing the PCR products. We successfully sequenced 138 samples from salt ponds and 136 from natural mangroves.

### Microsatellites genotyping

We tested primer pairs previously developed for the crabs *Uca mjoebergi* [45] and *Ucides cordatus* [46–47]. For each test, gel electrophoresis was performed to check DNA amplification. Seven primer pairs amplified the loci in *A*. *occidentalis*. The forward primer was labelled with a fluorescent dye (Table 1). A total volume of 12 μl, containing 1 μM of 2x multiplex mix (Qiagen, Hilden-Germany), 0.2 μM of primer mix, 2.5 μl of 10 mg/ml of BSA, and 2.4 μl of DNA extracts was used to amplify the microsatellite loci. The annealing temperature for the first four primers in Table 1 was 50.4°C for 90 s, while for all other primers it was 50.6°C for 90 s. The common PCR condition for both multiplex PCRs was: initial denaturation for 5 min at 95°C, denaturation of 30 s at 95°C for 31 cycles, and 72°C for 30 s, and final extension of 30 min at 60°C. Because of the differences in annealing temperatures we developed two multiplex PCRs, one containing of four primers and the other three primers. The PCR products for a few samples from different sites were selected and diluted 60 and 80 times. Based on two multiplex sets and the two dilutions, 1 μL of the diluted PCR products was mixed with 0.15 μL GeneScan 500 LIZ Size Standard and 8.85 μL of Hi-Di Formamide and then analysed on an ABI 3730 DNA Analyzer (Applied Biosystems) with a 50 cm capillary to measure the fragment size of the different microsatellite loci. One locus among the seven had a fragment size greater than 500 bp. The 80 times dilution resulted in clear fragment peaks compared to 60 times dilution. Therefore, the two multiplex PCRs were prepared in the same way as the previous one, but the locus with a fragment size of more than 500 bp was not included, because PCR did not work well as shown by presence of stutter bands and null alleles in many samples.

**Table 1:** Characteristics of primers used for microsatellites analysis in *Austruca* occidentalis from Tanzania, Western Indian Ocean. The superscripts a, b, and c at each locus indicate the source of each primer pair.

Locus	GenBank Accession number	Primer sequences (5’–3’)	Dye 5’ foward primer	Repeat motif	Allele size in this study	Allele size
P2D3^b^	FJ447550	F: CAACGACTTTAGGCCCACAC	6FAM	(TC)16	324–448	283–402
		R: TTGTATTGCAGACACGCTCC				
CAG7^b^	FJ447551	F: CCAGGATGTTATGAAGCTGGTC	ROX	(GA)22	324–338	232–278
		R: GATTTCTGCTGCCTCGTTTG				
CAC4^b^	FJ447547	F:AAGTGCGATAACCAAGGAGGCG	HEX	(AC)14(TC)15	259–291	242–300
		R:TGTGAGTTGGCTGTGTGATATGGC				
C361^a^	EU703141	F:CTCTTCACCACTTCACTCTTTGTCAGCC	ROX	(CTGC)5CTC(TCTG)6	336–448	323–370
		R:TGAGCCAGACAGGTAACTACAAAACGAGAC				
CT155^a^	EU703139	F: ACCGCTACACCAGCCATAAC	HEX	(GT)22	262–284	130–251
		R:TGGAAATGAAGACCAGAAAGG				
UsSSR26^c^	_	F:ATCTGGCATGAGTTTTCGTGT	6FAM	(GT)7	180–188	109
		R:TATTCTCCTCTGTAGCCCTGGA				
C359b^a^	EU703142	F: AAATAAAGCTCTGGACTATA CGACTTGTGC		(AAAG)6(GAGG)2GAAT(GAAA)2 AA(GAAA)2(GGAA)5GACAA GAA(AGGG)4(AAGG)3	>500	358–442
		R: AATAATGGTAATGTTACG TTCAGCCATCTC				

[47] ^**a**^, [48] ^**b**^, [49] ^**c**^.

### Mitochondrial DNA (mtDNA) and microsatellites analysis

The sequences were first edited by using the software Chromaspro v. 1.5 (Technelysium) and compared with sequences available in GenBank using the online software BLAST in order to confirm species identity. The presences of stop codons, which indicate sequencing errors or pseudogenes, was evaluated by using the program Squint Alignment Editor v. 1.02 [48]. A multiple alignment of the sequences was done by using CLUSTAL W [49] as implemented in the software MEGA 6 [50]. To determine the heterogeneity of haplotype distribution between salt pond sites and natural mangrove sites, a contingency *χ*^*2*^ table via a Monte Carlo simulation implemented in the software R: package coin version 1.1–2 [51] was used. This involved simulating *χ*^*2*^ with a p-value that was based on 2,000 replicates. The online FaBox 1.41 Collapse tool was used for collapsing the sequences to haplotypes. The statistical parsimony approach as implemented in the software TCS 1.21 [52] was used to investigate the relationship between haplotypes from salt pond sites and natural mangroves. Nucleotide and haplotype diversity, historical demography [53–54], as well as neutrality parameters [55–56], were calculated by using the software Arlequin v. 3.5.2.2 [57]. The within and among population differentiation was investigated by analysis of molecular variance (AMOVA) [58]. Hierarchical AMOVA was used to determine if salt farming influences the gene flow in *A*. occidentalis. Analysis of variance (ANOVA) was performed with the software R (Version 3.1.2) to determine differences in nucleotide and haplotype diversity, observed and unbiased heterozygosity and allelic richness between populations from salt ponds and natural mangroves. Genetic diversity, heterozygosity and allelic richness were treated as dependent variables and “type of habitat “(i.e. salt pond and natural mangrove sites) as an orthogonal and fixed factor. Before ANOVA tests all dependent variables were tested for normality using Shapiro-Wilk and homogeneity of variances using Levene’s test and Fisher's F- test as implemented in the software R (Version 3.1.2). The values of unbiased heterozygosity were normally distributed after Ln-transformed.

Scoring of alleles was done manually with GeneMarker (2.4.0; SoftGenetics, State College, PA, USA). All samples found to have missing values at three loci or could not be scored were removed from the data set. The final data set consists of 173 samples from salt ponds and 159 from natural mangroves. We used the software MICRO-CHECKER v. 2.23 [59] to test for the presence of null alleles, large allele dropout, or scoring errors. The software GenAlEx v. 6.5 [60] was used to test for departure of each locus from Hardy–Weinberg equilibrium (HWE), and the resulting P-values were adjusted by sequential Bonferroni correction. Analysis of molecular variance was conducted in Arlequin v. 3.5.2.2 [57]. The number of alleles, effective number of alleles, observed and unbiased estimate of heterozygosities as well as the inbreeding coefficient within populations (F_is_) were calculated by using the software GenAlEx: version 6.5 [60]. GenAlEx was used to perform principle coordinate analysis (PCoA) for pairwise F_st_-values obtained from sequences and microsatellite data. The software Fstat [61] was used to calculate the allelic richness per population.

The program MIGRATES v. 3.11.6 [62] was used to estimate mutation–scaled effective population size (Θ = 4N_e_μ) in microsatellite data, where N_e_ represents the effective population size and μ is the mutation rate per generation per locus. The same software was used to infer for the mutation-scaled migration rate M (M = m/μ) between populations from salt ponds and natural mangroves, where m represents migration rate per generation, and μ represents the mutation rate [63]. A Brownian mutation model was used. The initial runs were performed in three replicates, each consisting of one long chain with 50,000 recorded steps and 50 increments. The sampling parameter value and burn-in was 2,500,000 and 100,000, respectively, with an exponential prio distribution. The final runs consisted of one long chain of 50,000 recorded steps, 50 increments, four replicates, 2,500,000 sampled parameter values and a burn-in value of 100,000. The heating scheme was with multiple Markov chains and four static temperatures that started at 1, 1.5, 3, and 1,000,000 with exponential prio distribution. The software MIGRATE uses the coalescent approach to estimate the relative effective population size and asymmetric gene flow between pairs of populations over 1000s of years [64]. To estimate the recent migration patterns between population at salt ponds and natural mangroves sites, we used the software BAYESASS v. 3.0.3 [65], which uses a Bayesian approach. This software estimates migration rates over a short period of time, approximately 3–6 years [64]. Seven runs were first performed by changing the number of seeds (s = 10, 100, 250, 500, 600, 750 and 1000) in order to obtain a suitable convergence. The number of iterations was 3,000,000, of which 100,000 were burn-in, and the sampling frequency was 2000. Mixing parameters were 0.3 for allele frequencies, 0.3 for inbreeding coefficients, and 0.5 for migration rates. The final run consisted of the same mixing parameters and 750 numbers of seeds.

## Results

### Patterns of haplotypes and nucleotide diversities

**Fig 2:**
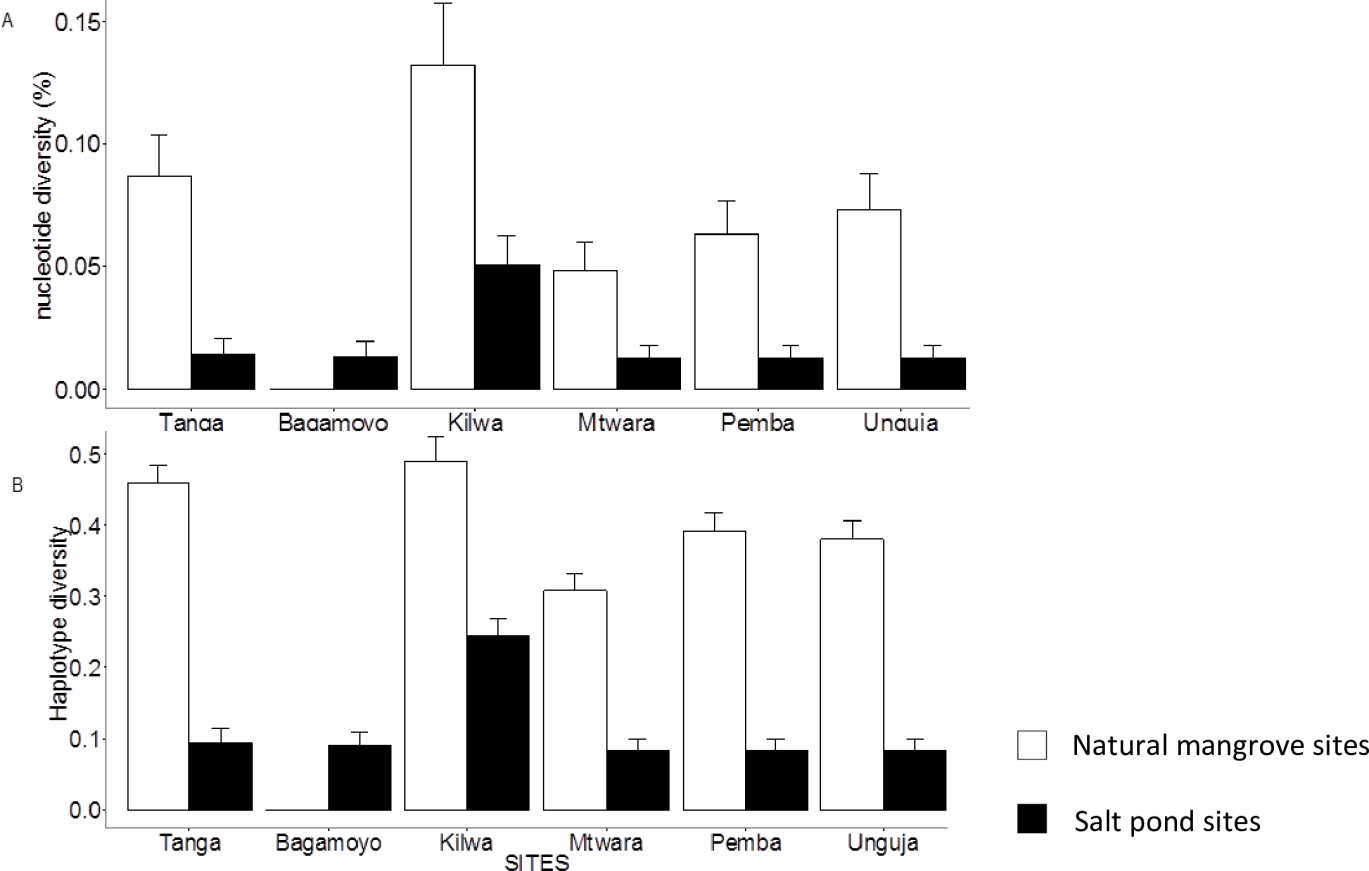
Genetic diversity in the fiddler crab *Austruca occidentalis* from natural mangroves (whitefilled bars) and mangroves at salt ponds (black filled bars) in Tanzania, Western Indian Ocean. A: Nucleotide diversity and B: haplotype diversity.

After aligning and trimming unreadable parts of sequences, we obtained an alignment of 674 bp length. All sequences are deposited at the European Nucleotide Archive (ENA) with the accession numbers LT703011-LT703284. Mitochondrial haplotype diversity for all populations was 0.23 (0 to 0.49), and nucleotide diversity 0.04% (0 to 0.08%) (Fig 2). Both, genetic diversity and the number of haplotypes were found to be consistently lower at salt pond sites compared to natural mangrove sites, with exception of the site Bagamoyo. The number of private haplotypes was lower at salt ponds sites compared to natural mangrove sites.

**Fig 3:**
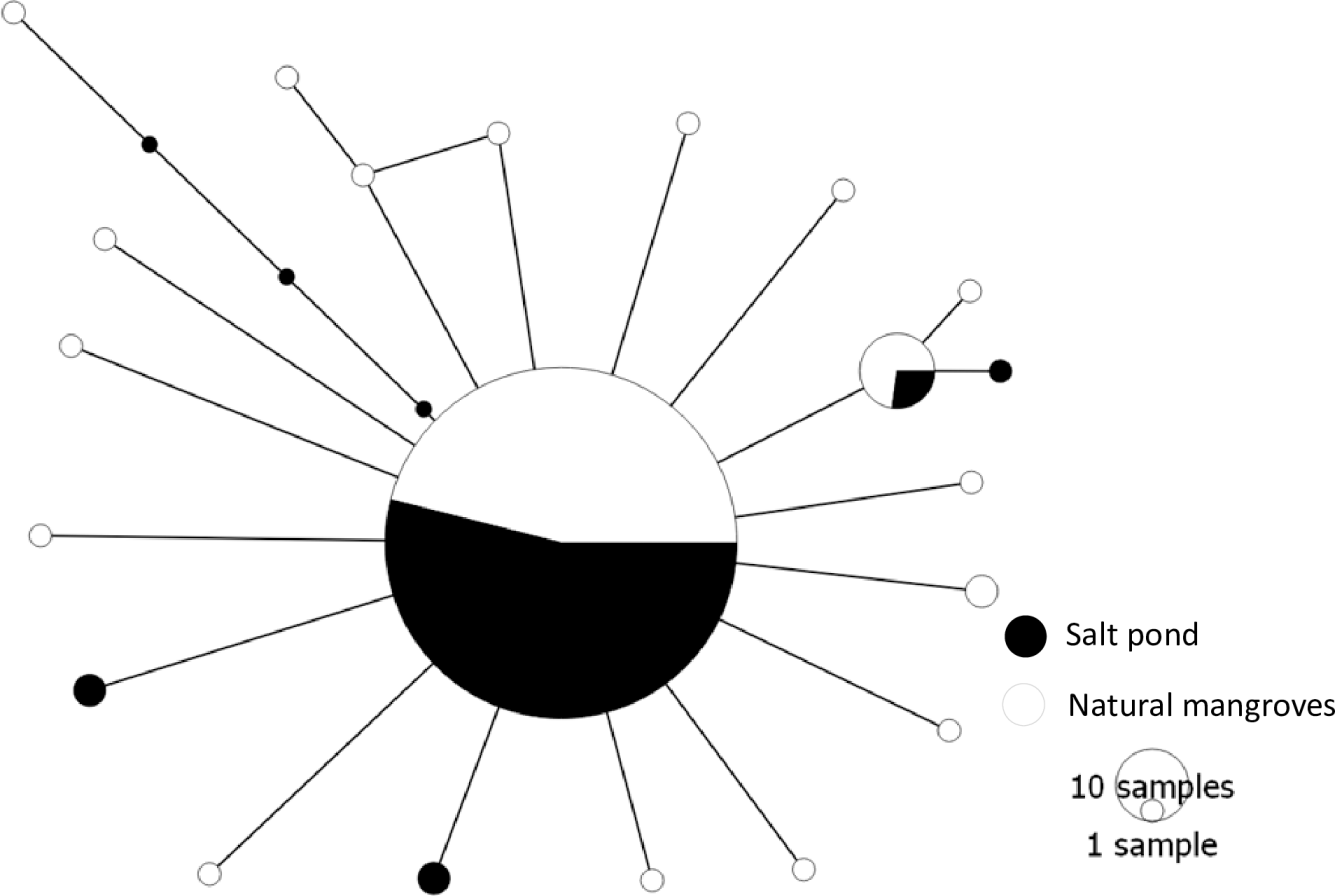
Haplotype network of partial mitochondrial cytochrome oxidase subunit I (COI) sequences from the fiddler crab *Austruca occidentalis* in Tanzania, Western Indian Ocean. The central circle in the haplotype network is representing 241 individuals. The size of other circles corresponds to the number of individuals as indicated in the right side of the haplotype network. The haplotype network indicates percentage of haplotypes from natural mangroves (white) and mangroves at salt ponds (black).

The total number of haplotypes recorded was 21. Only two haplotypes, the commonest, were shared between salt pond sites and natural mangroves. Most individuals at all sites had haplotype one (Fig 3). The minimum spanning network is displaying a star-like structure, with most rare haplotypes differing only by one mutational step from a central haplotype (Fig 3). The number of haplotypes at the different sites ranged from one to six, with most natural mangrove sites having higher numbers compared to salt pond sites (Table 2). The mean and range of nucleotide diversity for all populations was 0.04% and 0–0.08%, respectively. In general, haplotype and nucleotide diversity were lower in populations from the salt ponds (0.11, 0.02%) compared to natural mangroves sites (0.34, 0.07%) (Fig 2). The ANOVA test supported the differences in nucleotide (F = 6.50, P = 0.03) and haplotype diversity (F = 8.48, P = 0.02) between population from salt ponds and natural mangroves.

**Table 2:** Distribution of cytochrome oxidase subunit I (COI) haplotypes of the fiddler crab *Austruca occidentalis* in Tanzania, Western Indian Ocean at natural mangrove sites and salt pond sites; N: number of individuals; Nh: number of haplotypes at each site; HN: total number of haplotypes for natural mangrove sites; HS: total number of haplotypes for salt pond sites;.N and.S are the codes for natural mangrove and salt pond sites respectively.

				Haplotype																				
Sites	Codes	N	Nh	1	2	3	4	5	6	7	8	9	10	11	12	13	14	15	16	17	18	19	20	21
Tanga	TN	23	6	17	2	0	0	0	0	0	0	0	0	0	0	0	1	1	1	1	0	0	0	0
	TS	21	2	20	0	0	0	0	0	0	0	0	0	1	0	0	0	0	0	0	0	0	0	0
Bagamoyo	BN	24	1	24	0	0	0	0	0	0	0	0	0	0	0	0	0	0	0	0	0	0	0	0
	BS	22	2	21	1	0	0	0	0	0	0	0	0	0	0	0	0	0	0	0	0	0	0	0
Kilwa	KN	18	6	13	1	1	1	1	1	0	0	0	0	0	0	0	0	0	0	0	0	0	0	0
	KS	23	3	20	0	0	0	0	0	1	2	0	0	0	0	0	0	0	0	0	0	0	0	0
Mtwara	MN	24	4	20	2	0	0	0	0	0	0	1	1	0	0	0	0	0	0	0	0	0	0	0
	MS	24	2	23	0	0	0	0	0	0	0	0	0	1	0	0	0	0	0	0	0	0	0	0
Pemba	PN	23	4	18	2	0	1	0	0	0	0	0	0	0	1	1	0	0	0	0	0	0	0	0
	PS	24	2	23	1	0	0	0	0	0	0	0	0	0	0	0	0	0	0	0	0	0	0	0
Unguja	UN	24	6	19	1	0	0	0	0	0	0	0	0	0	0	0	0	0	0	0	1	1	1	1
	US	24	2	23	1	0	0	0	0	0	0	0	0	0	0	0	0	0	0	0	0	0	0	0
**Total**	**HN**	136	27	111	8	1	2	1	1	0	0	1	1	0	1	1	1	1	1	1	1	1	1	1
	**HS**	138	13	130	3	0	0	0	0	1	2	0	0	2	0	0	0	0	0	0	0	0	0	0

The mean observed heterozygosity for all populations was 0.565 ± 0.026 and the unbiased expected heterozygosity was 0.497 ± 0.013. The allelic richness and heterozygosity were consistently higher at natural mangrove sites compared to salt pond sites. The ANOVA detected differences for observed heterozygosity (F = 15.15, P = 0.003), unbiased expected heterozygosity (5.47, P = 0.04) and allelic richness (F = 10.29, P = 0.01) between populations from salt ponds and natural mangrove sites. The within population inbreeding coefficient ranged from –3.40 to 0.026, but all were not significant different from zero. The total number of alleles per locus was low to moderate, ranging between two and ten, with C361 having the highest and D2P3 the lowest number of alleles. Private alleles were recorded only in four populations from natural mangrove sites (Tanga, Bagamoyo, Kilwa and Mtwara) (Table 3). Locus P2D3 deviated from HWE for all populations from natural mangroves, except for Pemba and Tanga. This locus deviated also from HWE for the population from Bagamoyo salt ponds. The locus C361 deviated from HWE for three populations from natural mangroves (Tanga, Bagamoyo and Unguja). It also deviated from HWE for populations from Unguja salt pond sites. The locus CAG7 and CT155 deviated from HWE in populations from natural mangroves at Pemba and at Tanga salt pond sites, respectively. The locus C361 indicated presence of null alleles for the populations from Tanga and Bagamoyo. Other scoring errors such as stuttering and large allele dropout among the loci were not detected.

**Table 3:** Genetic diversity in the fiddler crab *Austruca occidentalis* from sites at natural mangroves and salt ponds in Tanzania, Western Indian Ocean based on microsatellites. N: sample size; Na: number of different alleles; Ae: mean effective number of alleles; I: Shannon's information index; Ho: observed heterozygosity; uHe: unbiased expected heterozygosity; PA: number of private alleles; F_*is*_: inbreeding coefficient, Ar: Allelic richness;.N and.S are the codes for natural mangrove and salt pond sites respectively.

Sites	Code	N	Na	Ae	I	Ho	uHe	PA	F_*is*_	Ar
Tanga	TN	25	3.67	2.09	0.87	0.53	0.49	0.17	-0.12	3.52
	TS	27	3.50	2.10	0.85	0.44	0.49	0.00	0.03	3.43
Bagamoyo	BN	29	4.17	2.01	0.88	0.55	0.50	0.33	-0.12	3.79
	BS	30	3.67	1.90	0.81	0.46	0.46	0.00	-0.01	3.42
Kilwa	KN	26	3.83	2.02	0.85	0.67	0.50	0.67	-0.37	3.44
	KS	30	3.67	1.86	0.77	0.50	0.45	0.00	-0.10	3.30
Mtwara	MN	30	4.17	2.04	0.90	0.69	0.50	0.50	-0.40	3.86
	MS	30	3.83	2.06	0.87	0.56	0.49	0.00	-0.16	3.49
Pemba	PN	24	4.00	2.41	0.99	0.66	0.57	0.00	-0.24	3.80
	PS	30	3.33	1.96	0.83	0.52	0.49	0.00	-0.10	3.14
Unguja	UN	25	3.83	2.22	0.90	0.66	0.52	0.00	-0.27	3.60
	US	26	3.67	1.96	0.85	0.51	0.50	0.00	-0.05	3.50

### Genetic population structure and demographic history

**Fig 4:**
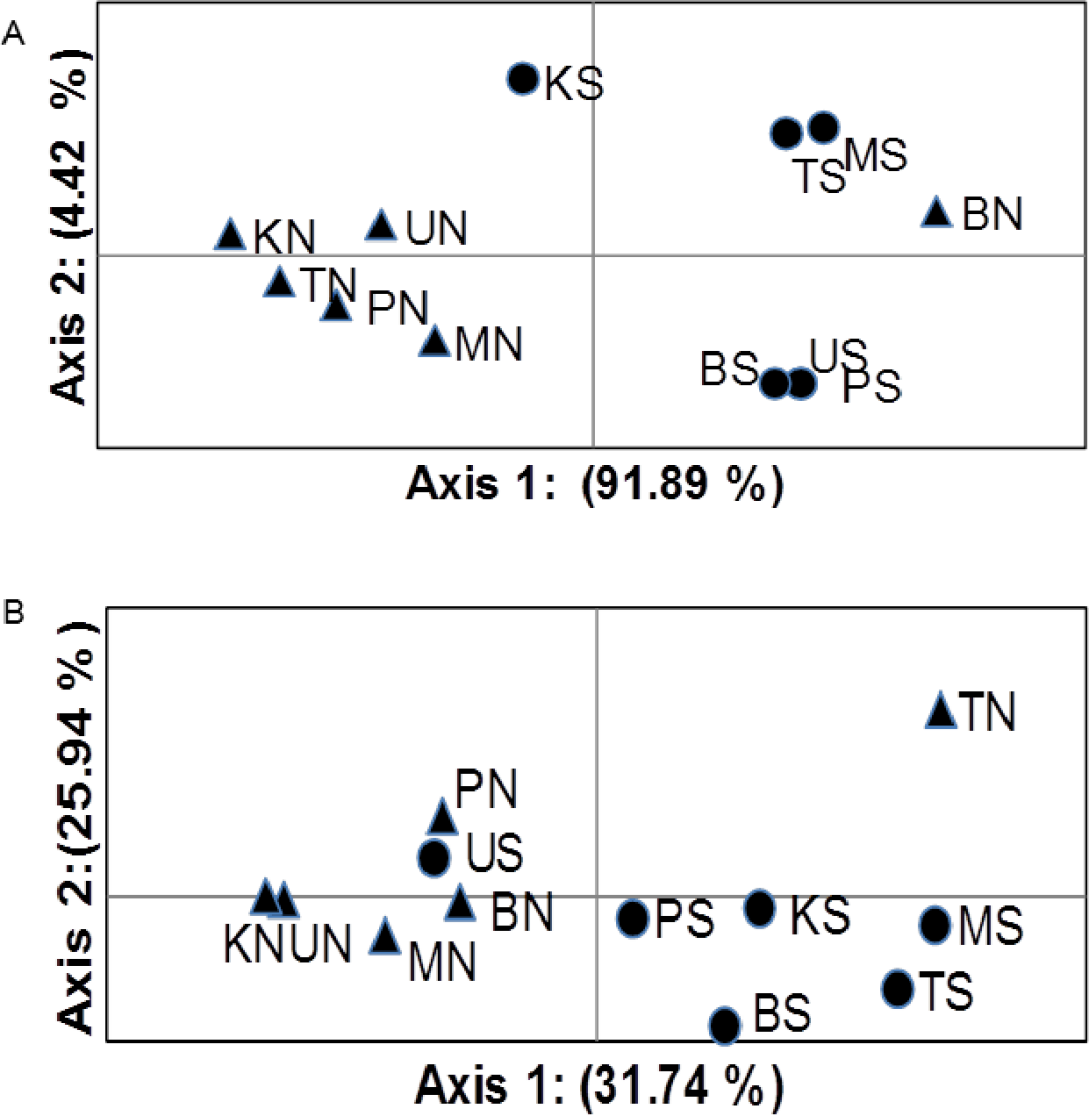
Principal coordinates analysis (PCoA) indicating population genetic differentiation based on pairwise F_st_-values in the fiddler crab *Austruca occidentalis* from Tanzania, Western Indian Ocean. : A) COI sequences and B) microsatellites. Circles represent salt ponds and triangles natural mangroves. (TN, BN, KN, LN, MN, PN, UN) and (TS, BS, KS, LS, MS, PS, US) represent Tanga, Bagamoyo, Kilwa, Lindi, Mtwara, Pemba and Unguja natural mangroves and salt ponds sites, respectively. The percentage of variation is mainly explained by the first axis. For sequences data is 91.89% and for microsatellites data is 31.74%.

The analysis of molecular variances of the COI sequences revealed a low but significant population differentiation (Overall F_st_ = 0.022, P < 0.05). The hierarchical (AMOVA) showed the existence of a low but significant genetic differentiation among samples from salt ponds and natural mangrove sites (F_ct_ = 0.033, P < 0.05). No differentiation was observed among populations from salt ponds (F_st_ = -0.009, P > 0.05) and natural mangrove sites (F_st_ = 0.008, P > 0.27) when they were analysed separately. Analysis through contingency chi-square tables revealed differentiations in haplotype distribution between subpopulations collected from mangroves at salt ponds and natural mangroves (χ2 = 383.73, p < 0.001). The pairwise Fst-values based on COI sequences for all populations were non-significant after applying sequential Bonferroni correction [66] (Table 4). The principal coordinate analysis (PCoA) indicates percentage of variations that is mainly explained by the first axis. For sequences data is 91.89% and for microsatellite data is 31.74% (Fig 4).

**Table 4:** Pairwise F_st_-values for cytochrome oxidase subunit I (COI) sequence data (bellow diagonal) and microsatellites (above diagonal) in the fiddler crab *Austruca occidentalis* from Tanzania, Western Indian Ocean; .N and .S are the codes for natural mangrove and salt pond sites respectively.

CODE	TN	TS	BN	BS	KN	KS	MN	MS	PN	PS	UN	US
TN	-	0.03*	-0.02	0.02*	-0.02	0.00	-0.03	0.03*	-0.02	-0.01	-0.02	-0.01
TS	0.06	-	0.02*	-0.01	0.05*	-0.00	0.02*	0.04*	0.06*	0.00	0.06*	0.04*
BN	0.13*	0.01	-	0.02	-0.00	0.01	-0.01	0.06*	0.00	-0.01	0.01	-0.01
BS	0.05	-0.02	0.00	-	0.04*	-0.01	0.02*	0.04*	0.06*	0.00	0.05*	0.03*
KN	-0.03	0.07	0.15*	0.07	-	0.01*	0.00	0.07*	-0.01	0.01	-0.01	-0.00
KS	0.02	0.01	0.06	0.01	0.02	-	0.01	0.03*	0.03*	-0.01	0.02*	0.02*
MN	-0.02	0.02	0.08	0.00	-0.01	-0.00	-	0.05*	-0.00	-0.00	0.01*	-0.00
MS	0.07*	-0.05	0	-0.02	0.09	0.02	0.03	-	0.06*	0.05*	0.06*	0.10*
PN	-0.03	0.04	0.10*	0.03	-0.03	0.00	-0.03	0.05	-	0.03*	-0.01	0.01
PS	0.06	-0.02	0	-0.05	0.08	0.02	0.01	-0.02	0.04	-	0.03*	0.03
UN	-0.02	0.03	0.09*	0.03	-0.02	-0.00	-0.02	0.04	-0.02	0.03	-	0.05
US	0.06	-0.02	0	-0.05	0.08	0.02	0.01	-0.02	0.04	-0.04	0.03	-

P < 0.05, Significant P-values are indicated by* and adjusted P-values after sequential Bonferroni are indicated by grey cells with*.

**Fig 5:**
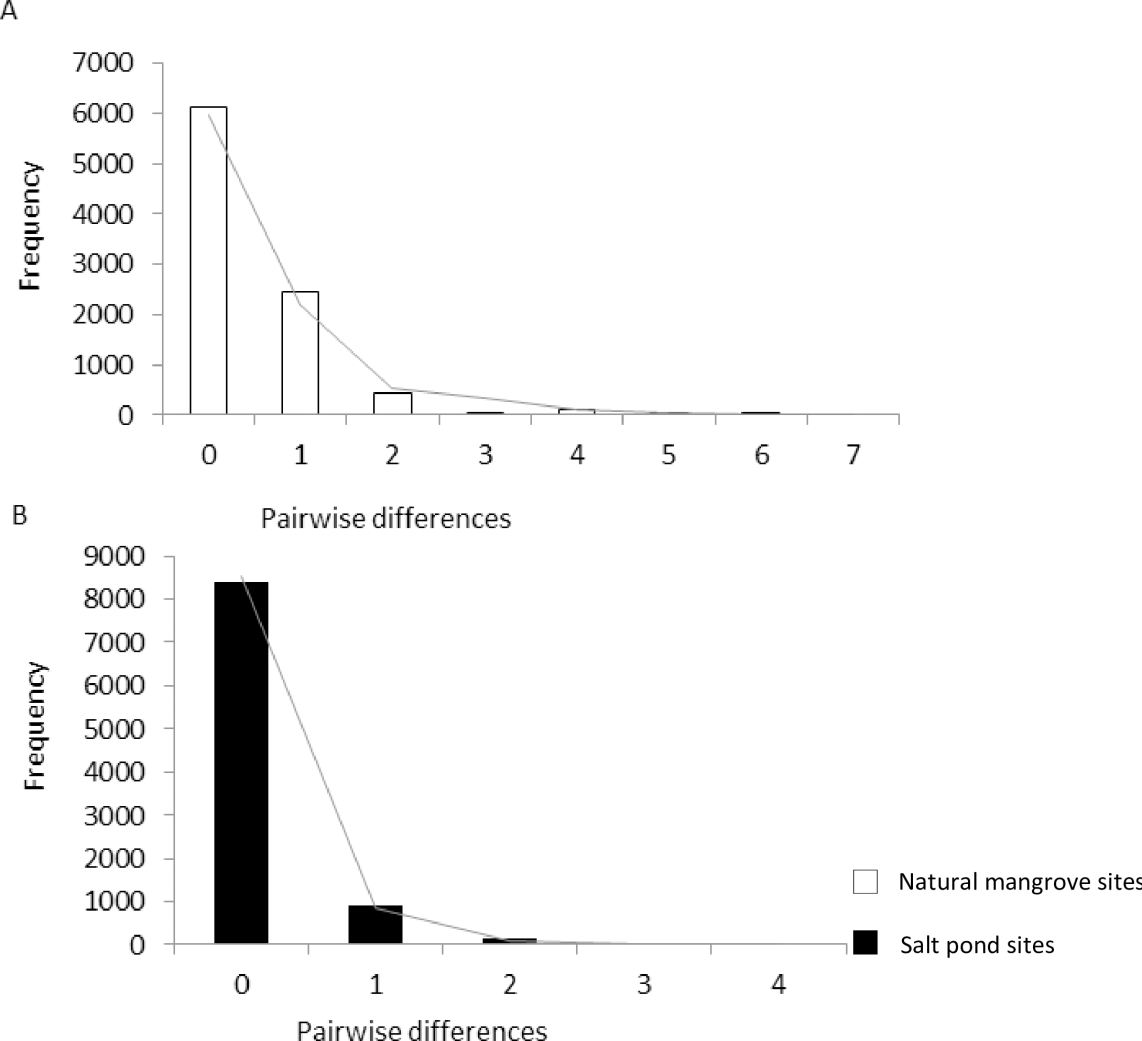
The observed (bars) and expected (line) frequency of pairwise differences of cytochrome oxidase subunit I sequences from the fiddler crab *Austruca occidentalis* in Tanzania, Western Indian Ocean. A. Populations from natural mangroves and B. populations from mangroves at salt ponds.

All Tajima’s D and Fu’s Fs test values were negative, and all significant P-values observed were from the populations of natural mangrove sites. Mismatch distribution analysis and Rogers’ test supported the hypothesis of sudden population expansion (Table 5). The mismatch frequency distribution curve was unimodal for both populations from salt ponds and natural mangrove sites (Fig 5).

**Table 5:** Demographic and neutrality parameters based on cytochrome oxidase I (COI) sequences from the fiddler crab *Austruca occidentalis* from Tanzania, Western Indian Ocean. SSD: sum of squared deviations, HRI: Harpending’s raggedness index, D: Tajima’s D and *Fs*: Fu’s Fs,.N and.S are the codes for natural mangrove and salt pond sites respectively.

Sites	Codes	SSD	HRI	D	*F*_*S*_
Tanga	TN	0.00	0.10	-1.27	-3.79**
	TS	0.00	0.66	-1.16	-0.92
Bagamoyo	BN	NA	NA	NA	NA
	BS	0.00	0.68	-1.16	-0.96
Kilwa	KN	0.01	0.13	-2.15**	-2.82**
	KS	0.00	0.37	-1.48	-0.83
Mtwara	MN	0.01	0.24	-1.49	-2.38**
	MS	0.00	0.70	-1.16	-1.03
Pemba	PN	0.00	0.17	-1.68*	-3.27**
	PS	0.00	0.70	-1.16	-1.03
Unguja	UN	0.00	0.16	-1.83*	-4.34**
	US	0.00	0.70	-1.1593	-1.03

*p < 0.05.

**p< 0.01.

Analysis of molecular variances of microsatellite data revealed a low but significant population differentiations (F_st_ = 0.019, P < 0.01; R_st_ = 0.055, P < 0.01). Hierarchal AMOVA results supported the hypothesis of genetic differentiations among populations at salt ponds and natural mangrove sites (F_ct_ = 0.02, P = < 0.01). A low but significant differentiation was observed among populations at salt pond sites based on microsatellite data (F_st_ = 0.024, P < 0. 05), but no significant differentiation was revealed among populations from natural mangroves (F_st_ = -0.002, P > 0.05). Some populations have significant pairwise F_st_-values adjusted through sequential Bonferroni correction (Table 4). Most of the populations from salt ponds had a lower effective population size than to natural mangrove populations. The main direction of migration based on MIGRATE and BayesAss was in most cases from salt pond sites to natural mangroves (Table 6).

**Table 6:** Estimated effective population size and migration rate based on microsatellite data using the programs MIGRATE (mean and 95% confidence interval) and BayesAss (mean and standard deviation) in the fiddler crab *Austruca occidentalis* from Tanzania, Western Indian Ocean. Θ: mutation-scaled population size; m: pairwise migration (± standard deviation);.N and.S are the codes for natural mangrove and salt pond sites respectively.

MIGRATE									BayesAss	
Θ					m				m	
Sites	Codes	Mean (%)	(2.5%,	97.5%)	direction	mean	(2.5%,	97.5%)	direction	Mean (%)
Tanga	TN	366	0.20	7.07	TN→TS	7.47	0.00	14.40	TN→TS	0.4 ± 0.8
	TS	223	0.77	3.63	TN←TS	7.20	0.00	13.33	TN←TS	1.1 ± 1.8
Bagamoyo	BN	1898	2.13	10.87	BN→BS	31.16	13.87	48.53	BN→BS	0.9 ± 1.4
	BS	222	0.83	3.60	BN←BS	43.04	31.47	52.53	BN←BS	2.1 ± 3.2
Kilwa	KN	1391	3.20	27.50	KN→KS	26.36	26.93	49.07	KN→KS	0.6 ± 1.0
	KS	297	1.83	4.07	KN←KS	155.05	95.73	140.53	KN←KS	0.6 ± 1.3
Mtwara	MN	295	1.60	4.23	MN→MS	20.00	1.60	24.80	MN→MS	0.6 ± 1.0
	MS	167	0.57	2.63	MN←MS	35.97	24.80	46.40	MN←MS	0.4 ± 0.8
Pemba	PN	238	0.77	4.03	PN→PS	71.82	0.00	8.53	PN→PS	0.3 ± 0.7
	PS	177	0.00	5.43	PN←PS	13.82	3.20	23.47	PN←PS	1.1 ± 1.8
Unguja	UN	222	0.77	3.73	UN→US	23.75	0.00	20.53	UN→US	0.7 ± 1.3
	US	603	0.83	8.07	UN←US	38.48	2.67	24.53	UN←US	0.6 ± 1.2

## Discussion

### Genetic diversity

Both markers used revealed low genetic diversity in most populations of *A*. *occidentalis* studied. Low genetic diversity for this species is also reported along the East African coast, with a haplotype diversity of 0.21 (0 to 0.68) and nucleotide diversity of 0.04% (0 to 0.13%) [30]. In that study, lower genetic diversity is associated with historical events of population expansion, following a period of lower effective population size resulting from a bottleneck effect or founder event. The low genetic diversity recorded in this study can be explained by the same factors. The crab *A*. *occidentalis* has the lowest genetic diversity of all mangrove crabs studied so far in the WIO [67]. Despite the low genetic diversity recorded in the present study, the population from natural mangroves had a higher haplotype diversity compared to populations from salt pond sites. We found consistent lower allelic richness in populations from salt ponds compared to natural mangroves. The populations at salt pond sites had a lower observed and expected heterozygosity than those at natural mangroves sites. The salt ponds and the natural mangroves for the most of the sites are not far from each other (4 to 18 km), therefore we assume that the observed differences cannot be due to differences of their geographic location. The differences observed in allelic richness and heterozygosity are therefore likely to be explained by environmental stress and unfavourable conditions caused by the salt farming activities. Lower genetic diversity in populations of *Littoraria subvittata* is observed at salt ponds in comparison to natural mangroves at the Tanzanian coast [68]. A recent study has also demonstrated the negative correlation between heavy metal concentrations in tissues of prawns and genetic diversity [69]. This implies that anthropogenic activities, such as salt farming in mangrove habitats of *A*. *occidentalis*, can negatively affect the genetic diversity of various marine species.

The loci C361 and P2D3 deviated from HWE mainly in populations from natural mangrove sites. The deviation of locus C361 from HWE may be explained by the effects of null alleles. A deficit of heterozygosity might be an indication for null alleles [70] that was detected only for population from mangroves at salt ponds of Tanga and natural mangroves of Bagamoyo. The deviations of these loci from HWE for other populations may therefore be explained by other factors, such as inbreeding. Lower allelic richness and genetic diversity is associated with unfavourable environmental conditions and environmental stress during the recruitment process [71]. The number of alleles and heterozygosity are also expected to decrease due to reduction of effective population size [72]. Most populations from salt pond sites had a lower effective population size compared to natural mangrove sites. The on-going clearing and selective logging of mangrove trees in these areas reduces the environmental complexity, alters microhabitats, and is likely to increase predation on this species. The loss and fragmentation of habitats by humans is suggested to be the source of reduction in population size and genetic diversity [73–74]. These factors might result in low dispersal potential and low connectivity, due to reduced potential for re-colonisation and gene interchanges with other sources [74].

Population bottlenecks in marine organisms can be caused by unsuitable environments and predation, which causes mortality during the larval recruitment processes [75]. The habitats of *A*. *occidentalis* at salt pond sites are subjected to human influence through clearing and selective logging of mangroves in order to establish salt ponds and construct water reservoirs. This might have contributed to unfavourable environmental conditions for larval recruitment, such as predation and extremes in local microclimate. Predators, including birds and fishes, are known to have influence on the population distribution of fiddler crabs [76]. Impact of predation pressure on the rate of evolution of age and size of the wild guppy *Poecilia reticulata* was revealed in a period of four to eleven years, or 6.9 to 18.1 generations [77], indicating that predation can have strong impacts on genetic variation of some species in a short time period.

### Population structure and patterns of demographic history

The analysis based on F-statistics is congruent to previous studies, indicating that this species has extensive gene flow along the East African coast [30, 67]. The star-like haplotype network is an indicator for a shallow genetic structure. The test for probability of heterogeneity via contingency *χ*^*2*^ tables indicated the presence of differentiations in haplotype distribution between populations from mangroves at salt ponds and natural mangroves. The population genetic differentiation is partly supported by the results of principal coordinate analysis (PCoA). However, all pairwise F_st_-values for mitochondrial data were not significant, while for microsatellite data some populations from salt ponds and natural mangroves sites were significantly different. Few significant values were also obtained between populations from mangroves at salt ponds (Table 4). Previous studies on this species have reported very low significance to non-significant F_st_-values among groups of populations [30, 67]. The differences between the pairwise F_st_-values based on mitochondrial and microsatellite data might be explained by the higher resolution and statistical power of microsatellite markers in detecting effects of contemporary events.

All populations from natural mangroves have significant negative Tajima’s D values, except the populations in Tanga and Mtwara, while all populations from salt pond sites have negative values that are not significant. In addition, all populations from natural mangroves have significant negative Fu’s Fs-values, but all populations from salt ponds have none-significant negative values. A negative significant value of Tajima’s D is usually an indicator of the presence of evolutionary forces that cause departures from the neutrality of the genetic marker, such as recent expansion of population size following the retraction event. A negative value of Fs is evidence of population expansion and is regarded as more powerful than Tajima’s D [56]. The non-significant negative Fu's Fs-values obtained for all populations from salt ponds could indicate constant sizes, which could mean that salt pond activities have been a reason for stagnant populations. Seven Indo-Pacific crab species, including *A*. *occidentalis*, show clear signals of a recent bottleneck. This bottleneck is linked to the reduction of population size due to historical event of Pleistocene period and mangrove loss or degradation by pollution [67]. The test of the sudden population expansion model by using the sum of square deviations (SSD) [54] was not significant. This implies that the data do not deviate from the model of expansion. The raggedness index [53] did not reject the hypothesis of the recent population expansion model for all populations. We obtained a unimodal mismatch distribution for all populations from natural mangroves and mangroves at salt ponds, suggesting recent population expansion following reduction or a bottleneck [54]. The excess heterozygotes observed might be indicating that these populations are currently expanding [78]. During low sea-level stand species were affected by population bottlenecks due to loss of suitable habitats, whereas at the end of last glacial period the rise of sea level resulted in populations expansions through re-colinisation [30, 79]. These glacial events are well document in high latitudes and islands [80]. However, it has been suggested that during the Pleistocene, the Indian monsoon repeatedly changed in intensity and phase, and it might have changed oceanic circulation in the WIO [81–82]. These events might have had impacts on the change in demographic patterns of many marine species. Mangrove ecosystems, which are the habitat for *A*. *occidentalis*, have been reported to have shrunk globally during the period of low sea-level stands [83]. Although all populations indicated sign of recent historical population expansion, the effective population size was found to be lower in populations from mangroves at salt ponds. Human disturbance, including habitat destruction through clearing and selective logging of mangrove trees, could have caused the decline of population size, resulting in the lower genetic diversity observed. Suitable habitat availability during settlement and recruitment influences effective population size [84–85]. Habitat fragmentation results in increased isolation of subpopulations and is always associated with decreases in population size [40]. Populations that suffer from reduction of size require proper conservation plans, because they are most likely to suffer from an increased risk of extinction [86].

## Conclusion

Both, mitochondrial and microsatellite markers confirmed that salt pond activities have impacted gene flow and genetic diversity, indicating that the on-going clearing and selective logging of mangrove trees have shaped dispersal patterns and local effective population sizes of *A*. *occidentalis*. Genetic diversity and allelic richness are indicators of the long-term potential for adaptability and persistence of a given species in the habitat [87–88]. The proper management option for mangrove sites at salt ponds is restoration of habitats for *A*. *occidentalis* through planting mangroves around the salt pond sites and removing the barriers such as dykes of abandoned salt ponds to enable water flow. Otherwise, if alteration of habitats through degradation and fragmentation continues, there is the possibility of driving many species to local extinction.
